# Case Report: Sequential Transarterial and Trans-Cortical Venous Embolization of a Mixed Pial-Dural AVM

**DOI:** 10.3389/fneur.2022.839016

**Published:** 2022-02-28

**Authors:** Kevin Yeboah, Mary-Ann Fares, Paul Hansen, Albert J. Yoo

**Affiliations:** Department of Neurointervention, Texas Stroke Institute, Dallas, TX, United States

**Keywords:** pial-dural AVM, transarterial embolization, transvenous embolization, dural AV fistula, facial arcade

## Abstract

Endovascular therapy is the primary treatment modality for dural arteriovenous fistulas. Pre-treatment angiographic evaluation of dural fistulas must rule out the presence of a mixed pial component or supply from pial-dural collaterals, as the pial supply must be closed before definitive occlusion of the draining vein to prevent iatrogenic rupture. In this report, we described a case of a mixed pial-dural arterial venous malformation (AVM), which was effectively treated with a sequential transarterial and trans-cortical venous embolization.

## Introduction

Endovascular therapy is the primary treatment modality for dural arteriovenous fistulas (dAVFs). The presence of a mixed pial component or supply from pial-dural collaterals is rare, however should be identified prior to treatment. In this report, we described a case of a mixed pial-dural arterial venous malformation (AVM), which was effectively treated with a sequential transarterial and trans-cortical venous embolization.

## Case Presentation

**Figure 1:**
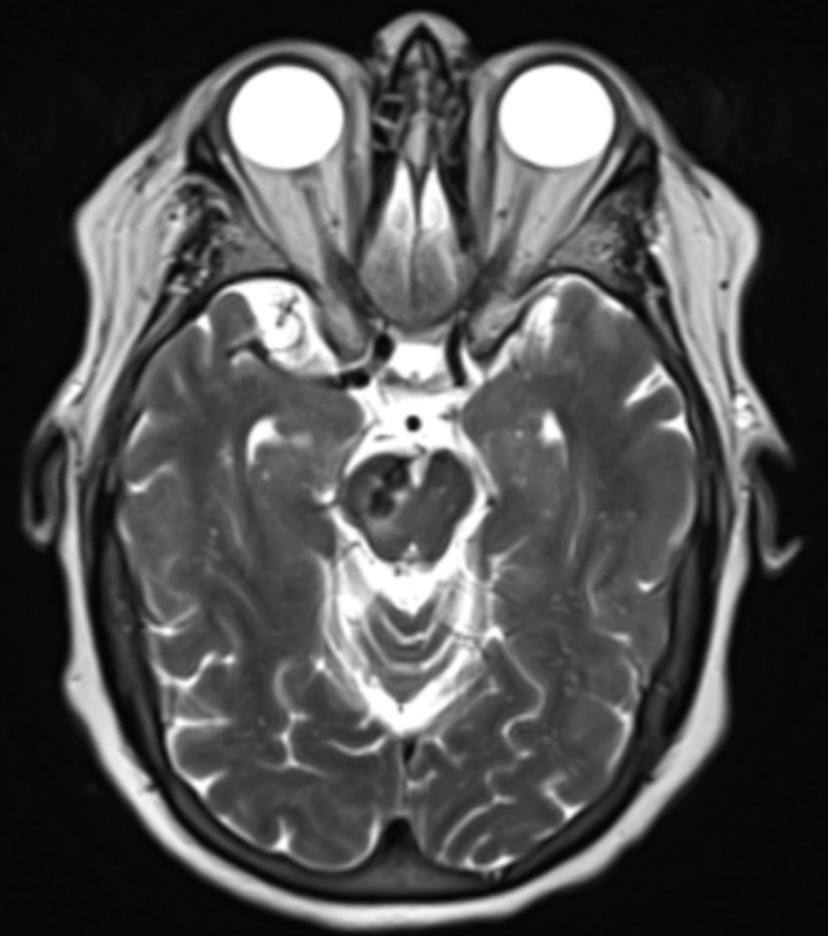
Axial T2 weighted image demonstrates bilobed flow void within the right cerebellar peduncle with surrounding edema and/or gliosis.

**Figure 2:**
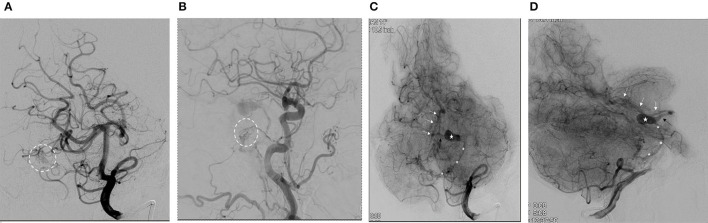
**(A)** Anterior-posterior oblique projection (arterial phase) demonstrates right superior cerebellar artery (SCA) supply to the arterial-venous malformation (AVM) nidus (dashed circle). **(B)** Lateral projection from right common carotid artery injection (arterial phase) demonstrates supply to the dural fistulous network (dashed circle) from the petrosal branch of the middle meningeal artery. **(C)** Anterior-posterior oblique projection from left vertebral artery injection (parenchymal phase) demonstrates the venous drainage from the AVM. (*, anterior pontomesencephalic vein; star, venous varix; black arrow, peduncular vein; white arrows, a basal vein of Rosenthal). **(D)** Lateral projection from left vertebral artery injection (parenchymal phase) demonstrates the venous drainage from the AVM. (*, anterior pontomesencephalic vein; star, venous varix; black arrow, peduncular vein; white arrows, a basal vein of Rosenthal).

We reported the case of a 73-year-old female with coronary artery disease, hypertension, and hyperlipidemia who underwent embolization of a symptomatic mixed pial-dural AVM. She presented with left-sided paresthesias, and MRI demonstrated a bilobed flow void in the right cerebral peduncle contiguous with a pial vein and with surrounding edema and/or gliosis (Figure 1). Cerebral catheter angiography revealed a Cognard type IV/Borden type III mixed pial-dural fistula along with the tentorial attachment to the right petrous apex. Arterial supply to a small nidus was from 2 branches of the right superior cerebellar artery (SCA) and the petrosal branch of the right middle meningeal artery (MMA) (Figures 2A,B). Venous drainage was into an isolated segment of the superior petrosal sinus which drained *via* a bridging vein into the anterior pontomesencephalic system. This in turn drained into the right basal vein of Rosenthal *via* the peduncular vein. At the junction of the anterior mesencephalic and peduncular veins was a posteriorly directed bilobed venous varix which measured 13 mm in length × 5 mm in width (Figures 2C,D). Eight weeks after clinical presentation, she underwent elective transarterial and transvenous embolization of the pial-dural AVM.

### Procedure

The procedure was performed under general anesthesia. Through a left radial artery approach, a Benchmark catheter was placed into the V3 segment of the left vertebral artery. Venous access was obtained transfemorally.

**Figure 3:**
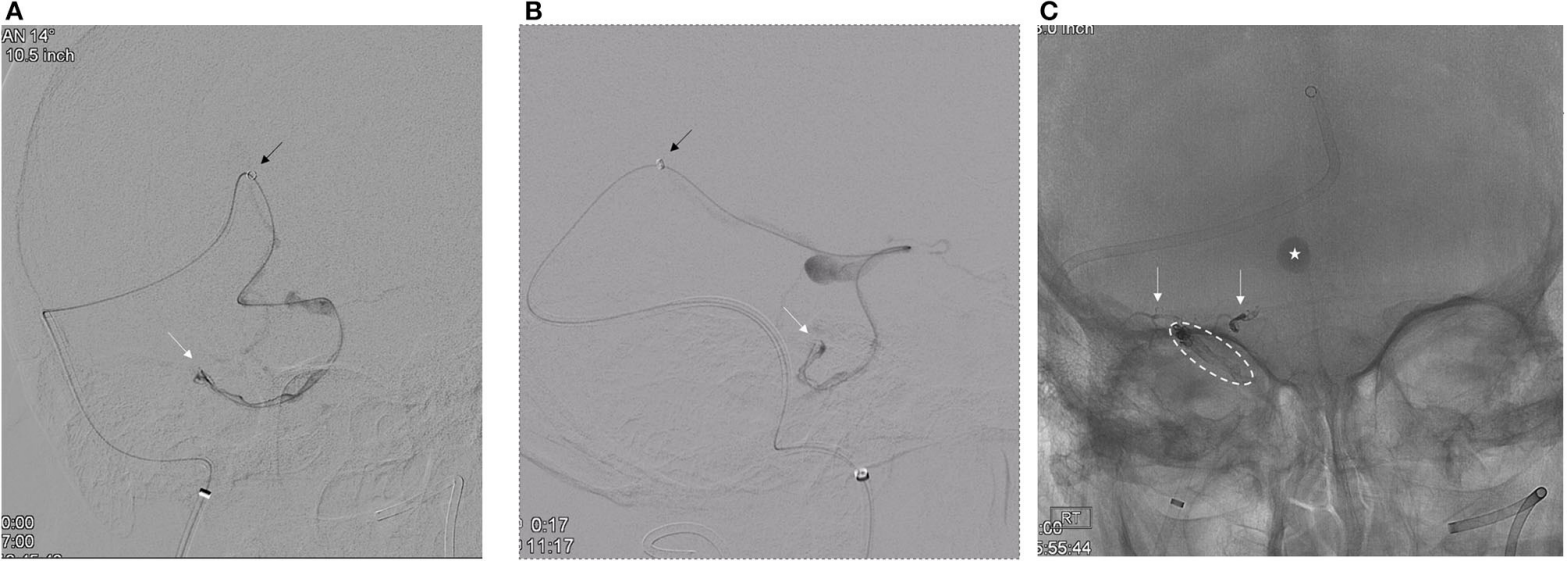
**(A,B)** Superselective microcatheter angiogram [**(A)**, AP oblique; **(B)**, lateral] confirms the microcatheter tip position (white arrow) within the recipient venous pouch. The intermediate catheter (black arrow) is in the distal vein of Galen. **(C)** White arrows delineate the Onyx casts within the arterial pedicles from the right SCA. Dashed oval circumscribes the coil and Onyx cast within the recipient venous pouch and the bridging vein. The star illustrates contrast stasis within the venous varix.

To minimize the risk of AVM rupture, transarterial closure of the pial supply from the right SCA was performed prior to transvenous occlusion of the recipient venous pouch. To ensure transvenous access for treatment, a microcatheter was placed into the venous pouch prior to transarterial embolization. A Ballast 80 cm 8F catheter (Balt, Irving, CA) was placed at the right jugular bulb. Through the Ballast, a Sofia Flow Plus 6F catheter (MicroVention, Aliso Viejo, CA) intermediate catheter was advanced over a Headway Duo 156-cm microcatheter and Synchro Select microwire into the right vein of Galen. To reach the recipient vein, the microcatheter was replaced with a Headway Duo 167 cm (MicroVention, Aliso Viejo, CA) microcatheter. Over a Traxcess microwire (MicroVention, Aliso Viejo, CA), this microcatheter was carefully advanced past the varix, through the anterior pontomesencephalic system, and into the venous pouch using a roadmap from the left vertebral artery injection (Figures 3A,B).

Then, through the Behnchmark catheter (Penumbra, Alameda, CA), a Sofia 5F catheter (MicroVention, Aliso Viejo, CA) was navigated into the basilar artery over a Headway Duo 156 cm, 167 cm (MicroVention, Aliso Viejo, CA) microcatheter and Synchro select microwire (MicroVention, Aliso Viejo, CA). The microcatheter was placed into the right SCA and advanced into one of the branches supplying the AVM nidus. The microcatheter was placed just proximal to the takeoff of a tiny pedicle supplying the lateral aspect of the nidus. Given the small caliber of the pedicle, it was decided to close the branch from this position with additional non-target embolization of supply to the superior and lateral aspect of the right cerebellum. A small amount of Onyx 18 (Medtronic, Minneapolis, MN) (<0.2 ml) was injected to achieve proximal closure of the small feeding pedicle, and the microcatheter was retrieved. Through the 5F SOFIA, another Headway Duo 156-cm microcatheter was advanced into a direct feeder supplying the medial aspect of the AVM nidus. A small amount of Onyx 18 (<0.2 ml) was injected achieving closure of the pedicle with partial penetration of the nidus. The microcatheter was retrieved.

After the closure of the pial supply to the AVM, a second Benchmark catheter was placed into the right common carotid artery where angiography confirmed arterial supply to the dural component of the AVM *via* the petrosal branch of the right MMA. To facilitate controlled Onyx embolization of the venous pouch, two Target 360 Nano coils (3 mm × 4 cm, 2 mm × 3 cm) were placed into the pouch through the Headway 167-cm microcatheter.

After the closure of the pial supply to the AVM, a second Benchmark catheter was placed into the right common carotid artery where angiography confirmed arterial supply to the dural component of the AVM *via* the petrosal branch of the right MMA. To facilitate controlled Onyx embolization of the venous pouch, two Target 360 Nano coils (3 mm × 4 cm, 2 mm × 3 cm) were placed into the pouch through the Headway 167-cm microcatheter. Then, ~0.3 ml Onyx 18 was injected until the recipient vein was occluded (Figure 3C). Progress was monitored with intermittent injections of the right CCA. After the closure of the AVM, the venous microcatheter was retrieved.

### Patient Outcomes

**Figure 4:**
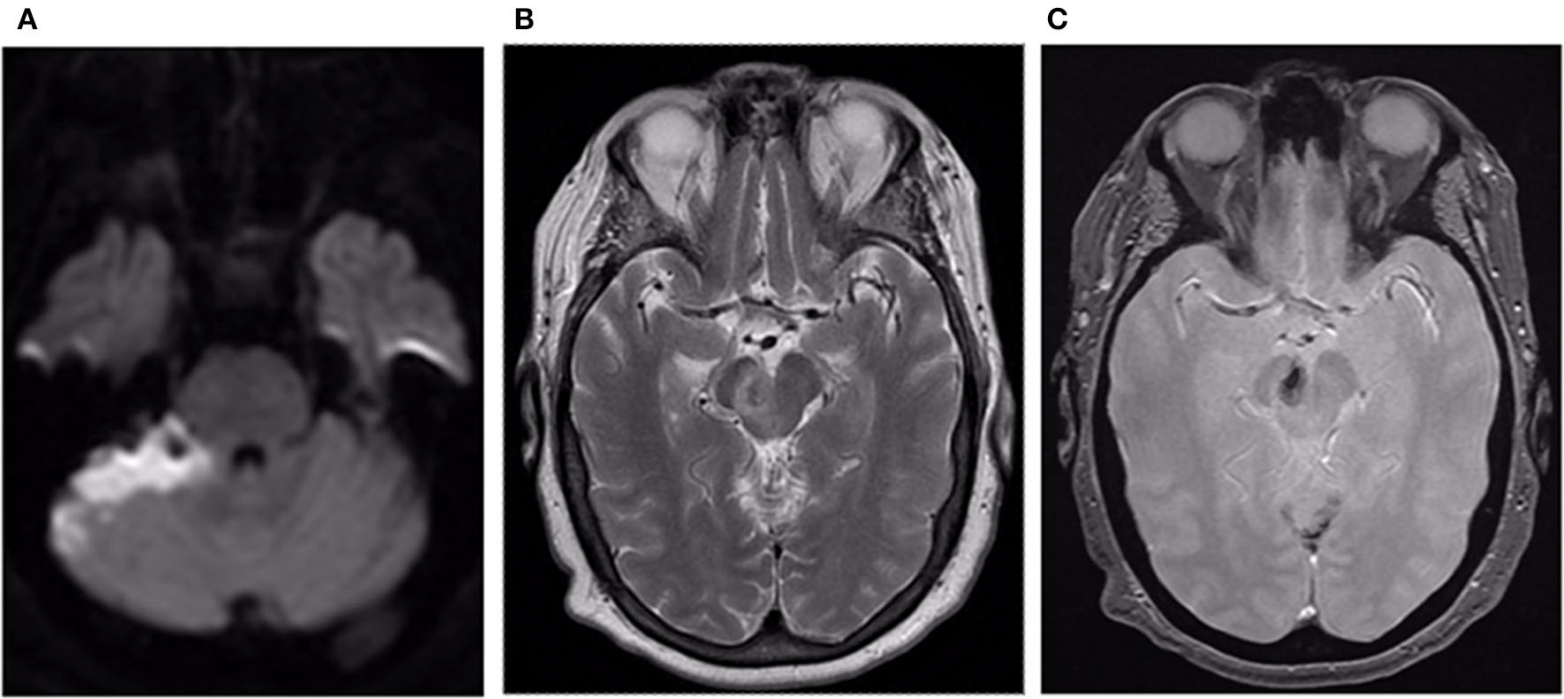
**(A)** Axial DWI image reveals acute infarct within the superior and lateral aspect of the right cerebellum. **(B)** Axial T2 weighted image reveals loss of the flow void within the venous varix in the right cerebral peduncle with surrounding edema. **(C)** Axial GRE image reveals thrombus within the venous varix.

Postoperatively, the patient was monitored in the Neuro ICU. She reported mild nausea, unsteady gait, and right-handed clumsiness. Follow-up MRI showed an expected infarct in the right superior and lateral cerebellar hemisphere and thrombosis of the venous varix (Figures 4A–C). She was discharged on day 7 after admission to inpatient rehab. At 3 months, she was living alone at home. She had residual mild balance difficulty but could ambulate independently with a walker. Her mRS score was 2.

## Discussion

Angiographic evaluation of dAVFs must rule out the presence of a mixed pial component or supply from pial-dural collaterals (e.g., the artery of Davidoff and Schechter), as endovascular treatment must occlude the pial supply prior to closure of the dural fistula. Because definitive treatment of dural fistulas requires closure of the recipient vein, the presence of a mixed pial component or pial collateral supply can result in AVM rupture due to outflow obstruction as seen with brain AVMs (1, 2).

Owing to the dural supply from the petrosal branch in this case, transvenous embolization was necessary to prevent injury to the facial arcade. The ability to traverse the cortical veins of the posterior fossa to reach the venous pouch illustrates the increased safety profile afforded by the current generation of highly compliant and deliverable intermediate catheters and microcatheters. Endovascular closure of the draining vein can be achieved using coils, liquid embolic agent, or a combination as in this case. Flow-directed microcatheters that can also deliver detachable coils are particularly useful for this application. Depending on the length and tortuosity of the cortical venous drainage, it is often not possible to achieve transvenous access to the fistula site, in which case a multimodal approach using radiosurgery or neurosurgery is necessary.

A unique feature of this case was the relative prominence of the venous varix which was disproportionate to the overall degree of venous ectasia. The size of the varix and its eloquent location with the cerebral peduncle contributed to the patient's neurological deficit in the absence of rupture.

## Conclusion

Endovascular therapy is the mainstay of treatment for dAVFs. Mixed pial-dural AVMs are rare lesions that are important to identify because treatment must target the pial supply prior to definitive closure of the draining vein to prevent iatrogenic rupture.

## Data Availability Statement

The original contributions presented in the study are included in the article/supplementary material, further inquiries can be directed to the corresponding author(s).

## Ethics Statement

Ethical review and approval was not required for the current study in accordance with the local legislation and institutional requirements. Written informed consent was not required for the current study in accordance with the local legislation and institutional requirements. Written informed consent was obtained from the individual(s) for the publication of any potentially identifiable images or data included in this article.

## Author Contributions

All authors listed have made a substantial, direct, and intellectual contribution to the work and approved it for publication.

## Conflict of Interest

The authors declare that the research was conducted in the absence of any commercial or financial relationships that could be construed as a potential conflict of interest.

## Publisher's Note

All claims expressed in this article are solely those of the authors and do not necessarily represent those of their affiliated organizations, or those of the publisher, the editors and the reviewers. Any product that may be evaluated in this article, or claim that may be made by its manufacturer, is not guaranteed or endorsed by the publisher.
